# [^68^Ga]Ga-FAPI PET/CT in brain tumors: comparison with [^18^F]F-FDG PET/CT

**DOI:** 10.3389/fonc.2024.1436009

**Published:** 2024-09-06

**Authors:** Ya Liu, Haoyuan Ding, Jianpeng Cao, Guangfu Liu, Yue Chen, Zhanwen Huang

**Affiliations:** ^1^ Department of Nuclear Medicine, The Affiliated Hospital of Southwest Medical University, Luzhou, Sichuan, China; ^2^ Nuclear Medicine and Molecular Imaging Key Laboratory of Sichuan Province, Luzhou, Sichuan, China; ^3^ Institute of Nuclear Medicine, Southwest Medical University, Luzhou, Sichuan, China; ^4^ Department of Nuclear Medicine, Second Affiliated Hospital of Chengdu Medical College (China National Nuclear Corporation 416 Hospital), Chengdu, Sichuan, China

**Keywords:** PET/CT, 18F-FDG, 68Ga-FAPI, brain, tumor

## Abstract

**Purpose:**

To investigate the feasibility of [^68^Ga]Ga-FAPI PET/CT in brain tumor imaging and to compare it with [^18^F]F-FDG PET/CT.

**Methods:**

25 patients with MRI-suspected brain tumors were included in the study. They underwent whole body [^18^F]F-FDG PET/CT and [^68^Ga]Ga-FAPI PET/CT and brain scans. The target-to-background ratio (TBR) of brain tumors was calculated with the background of surrounding normal brain tissues uptake. The SUVmax and TBR of [^18^F]F-FDG PET/CT and [^68^Ga]Ga-FAPI PET/CT were compared. Additionally, the correlation between the uptake of the tracer by lesions with the greatest diameter of the lesion, the breadth of the oedema band, and the enhancement scores of the MRI enhancement scans was analyzed.

**Result:**

[^68^Ga]Ga-FAPI PET/CT was superior to [^18^F]F-FDG PET/CT for lesion detection, especially for brain metastases. Among gliomas, only high-grade gliomas uptake [^68^Ga]Ga-FAPI. Compared with [^18^F]F-FDG PET/CT, [^68^Ga]Ga-FAPI PET/CT had a lower SUVmax but a significantly better TBR. On [^68^Ga]Ga-FAPI PET/CT, the TBR may be associated with brain tumor blood-brain barrier disruption.

**Conclusions:**

[^68^Ga]Ga-FAPI PET/CT is a promising imaging tool for the assessment of brain tumors. Lack of physiological uptake of [^68^Ga]Ga-FAPI in normal brain parenchyma results in high TBR values, leading to better visualization of lesions and contributing to subsequent targeted therapy studies.

**Advances in knowledge:**

Clinical utility of [^68^Ga]Ga-FAPI PET/CT in brain tumors remains unclear, and there aren’t many similar studies in the literature. We evaluated the role of [^68^Ga]Ga-FAPI PET/CT in diagnosing brain tumors.

## Introduction

Brain tumors include primary brain tumors and brain metastases. According to the Global Cancer Statistics 2020 (1), primary brain tumors make up 1.6% of all malignant tumors. Although the incidence is low, tumor-related complications are more severe and treatment modalities are limited, making them one of the most aggressive cancers (2). Approximately 8.5-9.6% of cancer patients will develop brain metastases (3), which portend a poor prognosis for patients, with a survival rate of only 1-2 months without treatment. Early diagnosis and treatment can improve patient survival rate to some extent. Magnetic resonance imaging (MRI) is the main approach to diagnosing brain tumors (4), but its value in brain tumor grading, prognosis, treatment planning, and evaluating efficacy or suspected recurrence is limited (5).

[^18^F]F-FDG PET/CT assessment of intratumoral glucose metabolism is currently the most commonly used molecular imaging strategy. However, the physiological uptake of glucose by normal brain tissue may mask intracranial lesions and the impact of blood glucose levels on imaging (6, 7). ^18^F-fluoroethyl-L-tyrosine ([^18^F]F-FET) PET/CT can also be applied to brain tumors (8). Normal brain tissue does not take up [^18^F]F-FET (9), resulting in a clearer tumor profile, but its clinical application is limited by its low renal clearance, prolonged retention in the blood, and low specificity (10, 11). There are many other amino acid PET-tracers recommended as molecular imaging agents for brain tumor imaging (10, 12–16). However, amino acid tracers are usually labeled by [^11^C]C and [^18^F]F (17). The half-life of [^11^C]C is very short, only 20 minutes (18), and the condition of [^18^F]F labeling these tracers is difficult to label successfully (19, 20), which result in high cost and limited clinical application.

Recently, the tumor microenvironment has been increasingly studied (21). Cancer-associated fibroblasts (CAFs) are the major cell subpopulation in the tumor stroma, and CAFs in most epithelial tumors exhibit overexpression of the type II transmembrane serine protease known as fibroblast activating protein (FAP) (22). ^68^Gallium-labeled FAP inhibitor ([^68^Ga]Ga-FAPI) is capable of specifically binding FAP and has emerged as a potential tumor imaging agent (23, 24).

In recent years, [^68^Ga]Ga-FAPI PET/CT has become the focus of nuclear medicine worldwide, and its application prospects in the diagnosis of tumor and non-tumor diseases are outstanding (25), especially in gastrointestinal tract, hepatobiliary system, peritoneal tumors (26–28). No adverse effects of [^68^Ga]Ga-FAPI PET/CT have been reported in either animal or human studies (29–31). And [^68^Ga]Ga-FAPI is easy to obtain and inexpensive (32), does not require any preparation before examination, and has a good tumor background ratio in various cancers, [^68^Ga]Ga-FAPI PET/CT becomes a test almost comparable to [^18^F]F-FDG, and is widely used in a variety of tumor and non-oncologic diseases (33). Its application in brain tumor imaging is also worth looking forward to.

Previous histopathological studies (34) have shown increased expression of FAP in glioblastoma, especially in the mesenchymal cell subtype, while it is rarely expressed in normal brain tissue (34). Another work showed that FAP promotes parenchymal invasion and epithelial-to-mesenchymal transformation in glioblastoma (35). FAP is also considered a potential therapeutic target for glioblastoma as well as a potential target for therapeutic drug delivery (36). Some studies have already reported increased expression of FAP in gliomas (37, 38). For tumors of epithelial origin, [^68^Ga]Ga-FAPI is superior to [^18^F]F-FDG in the detection of brain metastases in systemic assessment (39, 40). Therefore, [^68^Ga]Ga-FAPI may have great potential for application in patients with brain metastases. [^18^F]F-FDG is a tumor imaging agent commonly used in clinical practice (41). When studying the novel tracer [^68^Ga]Ga-FAPI, we chose it as a control group reference to reflect the advantages and disadvantages of the new PET molecular probe compared with the traditional probe, and at the same time make up for the uncertainty of [^68^Ga]Ga-FAPI in the systemic lesion evaluation of subjects (41, 42). So, we report the [^68^Ga]Ga-FAPI PET/CT imaging results from primary brain tumors and brain metastases. We additionally compare these findings to [^18^F]F-FDG PET/CT results.

## Materials and methods

### Participants

25 patients with brain lesions who underwent [^18^F]F-FDG (fluorodeoxyglucose) PET/CT for systemic assessment were enrolled in the [^68^Ga]Ga-FAPI solid tumor clinical trial (AHSWMU-2020-035) approved by the Hospital Ethics Committee (Clinical trial registration No.: ChiCTR2100044131). All participants provided written informed consent. An interval of no more than one week was employed between [^18^F]F-FDG PET/CT and [^68^Ga]Ga-FAPI PET/CT imaging. Patients eligible for inclusion were individuals who: (a) were 18+ years of age; (b) had suspected brain tumors on MRI at our institution between January 2021 and December 2022; (c) consented to undergo [^18^F]F-FDG PET/CT and [^68^Ga]Ga-FAPI PET/CT for the evaluation of primary or metastatic brain tumors; and (d) provided written informed consent for participation as per the guidelines of the study protocol. Patients were excluded if they were: (a) pregnant women; (b) already undergoing treatment before [^18^F]F-FDG PET/CT and [^68^Ga]Ga-FAPI PET/CT; (c) unable to lie flat for more than 30 minutes; (d) suffering from severe hepatic or renal impairment; or (e) suffering from brain herniation or confusion.

### Reference standard

For primary tumors, histopathological analysis of biopsy or excision specimens is the basis for definitive diagnosis. The pathological classification was referred to the 2007 WHO classification of tumors of the Central Nervous System (43). Unfortunately, there was a lack of information on IDH-mutant and IDH-WT. If the primary lesion is found on PET/CT, encephalopathy is defined as brain metastasis.

For the lung metastasis, of the 9 patients with lung cancer, 3 had adenocarcinoma and 2 had small cell lung cancer. Since there were already multiple metastases in the whole body at the time of discovery, some lung cancers were only confirmed as malignant tumors by biopsy, and there was no specific immunohistochemical classification, so they were not reflected in the paper.

### Preparation of radiopharmaceuticals

The Coincidence [^18^F]F-FDG synthesis module (FDG-N, PET Science & Technology) was used for standard [^18^F]F-FDG preparation. DOTA (1,4,7,10-tetraazacyclododecane-1,4,7,10-tetraacetic acid)-containing FAPI-04 was obtained from MedChemExpress. [^68^Ga]Ga-FAPI radiolabeling and purification were performed as reported previously. Radio-high-performance liquid chromatography confirmed that the resultant [^68^Ga]Ga-FAPI exhibited > 98% radiochemical purity. The final sterile [^18^F]F-FDG and [^68^Ga]Ga-FAPI preparations were pyrogen-free.

### PET/CT image acquisition

Before [^18^F]F-FDG PET/CT, patients were fasted for a minimum of 6 h to ensure blood glucose was within the standard range of 3.9-6.1 mmol/L. No special preparation was taken before [^68^Ga]Ga-FAPI PET/CT examination. The intravenous doses were 3.7 MBq/kg for [^18^F]F-FDG and 1.8-2.2 MBq/kg for [^68^Ga]Ga-FAPI (44). PET/CT (uMI780, United Imaging Healthcare) imaging was performed from the skull base to the upper thigh at 60 ± 10 min after injection, covering 6-8 beds (3 mins/bed) for torso acquisition and one bed (5-8 minutes) for dedicated head collection. Acquisition parameters: 120 kV, 120 mAs, 3.00 mm thickness. Lesion localization and correction for PET attenuation were performed using CT data. Sagittal, coronal, and cross-sectional PET and PET/CT images were generated by using an ordered subset expectation maximization algorithm (2 iterations, 20 subsets) for PET data reconstruction.

### PET/CT image review

To avoid bias, [^18^F]F-FDG PET/CT and [^68^Ga]Ga-FAPI PET/CT were retrospectively analyzed independently by two experienced nuclear medicine physicians (H.D and Z.H, respectively exhibiting 10 and 15 years of nuclear medicine experience). In PET/CT images, focal tracer uptake by the authorities was considered positive for uptake when it was greater than the background. Focal areas were considered negative when the uptake could not be differentiated from the adjacent background tissue. Discussion and consensus were used to resolve any disagreements. The lesion site, number, and SUVmax of each lesion were recorded, using the surrounding normal tissue associated with each lesion as the background. The target background ratio (TBR) was the lesion SUVmax divided by the background SUVmax. Inspired by previous studies (45–48), we set the background of TBR as contralateral normal brain tissue. Patients were classified into those with primary brain tumors and brain metastases based on the presence of other primary tumors on PET/CT. These classifications were confirmed by subsequent pathological findings and follow-up. In patient-based analyses, the highest SUVmax and TBR was selected to compare.

### MRI image review

MRI images were analyzed by an experienced radiologist. 7 patients underwent MRI with contrast and 18 underwent MRI without contrast. Lesion numbers and locations were recorded. For MRI with contrast, the degree of T1 serial enhancement, whether there was edema, and the maximum diameter of the lesion were recorded for each lesion. If there was no significant enhancement, a score of 0 was recorded. The degree of reinforcement was based on the medulla oblongata. If the degree of enhancement was comparable to or lower than that of the medulla oblongata, a score of 1 was recorded. If the degree of enhancement was greater than that of the medulla oblongata, a score of 2 was recorded. If oedema was present, the maximum diameter of the edematous zone from the edge of the lesion was recorded in cm. A value of 0 cm was recorded in the absence of edema.

### Statistical analysis

SPSS software 22.0 (IBM) was used for statistical analyses. Normally distributed data were reported as means ± standard deviations and other measures were expressed as medians. SUVmax and TBR values for all lesions were compared with the paired samples Wilcoxon signed-rank test. Differences between primary brain metastases and brain metastases on PET/CT were compared using the U test. Spearman correlation analyses were used to analyze correlations between lesion contrast uptake and MRI enhancement scoring, edema band width, and maximum lesion diameter. P < 0.05 was deemed statistically significant.

## Results

### Participant characteristics

A total of 25 patients (10 males and 15 females; median age, 57 years; range, 37-81 years; mean, 56.96 ± 10.6 years) were studied. All subjects were able to tolerate [^68^Ga]Ga-FAPI and [^18^F]F-FDG PET/CT. All patients’ vital signs (blood pressure/heart rate/body temperature) remained within normal ranges before, during and after the examination. None of the patients reported anything unusual. Of the 25 patients, 13 patients had primary brain tumors as confirmed by pathology after surgery, while 12 patients had primary foci identified on PET/CT and brain lesions defined as brain metastases. All patients underwent brain MRI as a standard imaging approach. Specific patient features are shown in **Table 1**.

**Table 1:** Patient characteristics.

Total patients	n=25
Median age	57
Sex	
Female	15
Male	10
KPS	
Median (range)	80 (40-100)
Primary brain tumors	13
gliomas	9
lymphomas	2
ependymoma	1
meningioma	1
Brain metastases	12
metastasis of lung cancer	9
metastasis of ovarian cancer	1
metastasis of thyroid cancer	1
metastasis of melanoma cancer	1

### Comparison of [^68^Ga]Ga-FAPI and [18F]F-FDG PET/CT detection rates

In evaluating these 25 patients with 40 total lesions as detected by MRI, [^68^Ga]Ga-FAPI PET/CT outperformed [^18^F]F-FDG PET/CT as a tool for lesion detection (30/40, 75% VS. 25/40, 62.5%, P=0.227). In the 13 patients with primary tumors with a total of 18 lesions, the [^68^Ga]Ga-FAPI PET/CT detection rate was consistent with that of [^18^F]F-FDG PET/CT (15/18, 83.3%). In the 12 patients with metastatic tumors with a total of 22 lesions, the [^68^Ga]Ga-FAPI PET/CT detection rate for metastatic lesions (15/22, 68.2%) was superior to [^18^F]F-FDG PET/CT (10/22, 45.5%) (P=0.227). [^68^Ga]Ga-FAPI exhibited higher detection rates for primary brain tumors than for brain metastases (83.3% *vs*. 68.2%, P=0.27) and also in [^18^F]F-FDG PET/CT (83.3%% *vs*. 45.5%, P=0.01).

### Focal-based analysis

**Figure 1:**
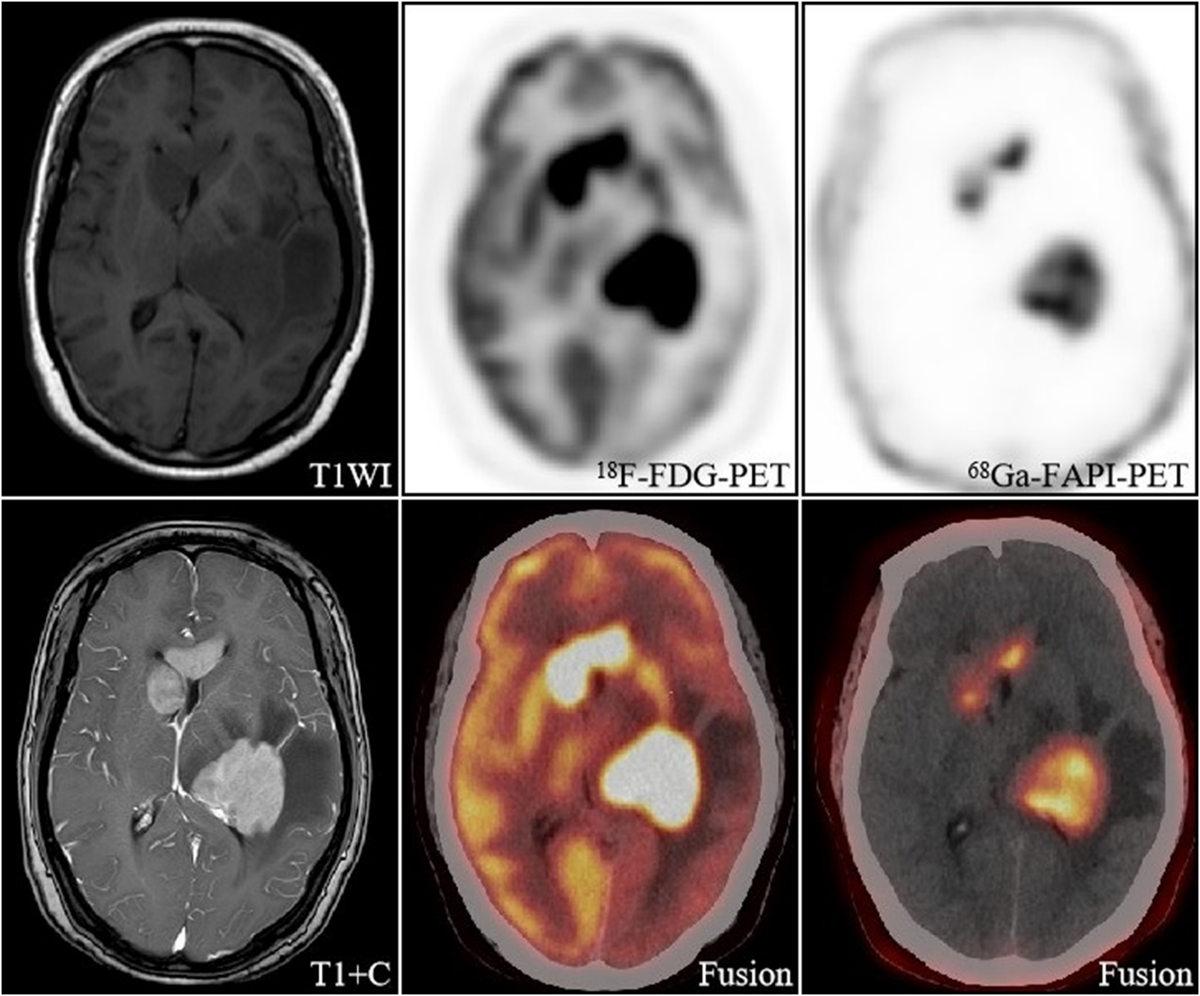
A 57-year-old woman, with a final diagnosis of intracranial diffuse large B-cell lymphoma. The intracranial lesions had a high uptake of [^18^F]FDG (SUVmax 8.5-32.1) and a much lesser degree and extent of uptake of [^68^Ga]FAPI than [^18^F]FDG, but still had a high TBR (67.5-78) due to a low background.

Among 13 patients with primary brain tumors, a total of 18 lesions were identified by imaging and confirmed by pathology. In [^68^Ga]Ga-FAPI PET/CT, the SUVmax was less than that of [^18^F]F-FDG PET/CT in all lesions (P < 0.001) (**Table 2**). Since normal brain tissue does not take up [^68^Ga]Ga-FAPI, the median TBR for [^68^Ga]Ga-FAPI PET/CT scans was 45, which was significantly better than that for [^18^F]F-FDG PET/CT scans of 1.9 (P < 0.001). Among the primary brain tumors, there were 9 lesions in 7 patients with high-grade glioma (5 of grade IV and 2 of grade III), and 3 lesions in 2 patients with low-grade glioma. Uptake of [^68^Ga]Ga-FAPI was observed in high-grade gliomas, with SUVmax values ranging from 0.5-6 and TBR values ranging from 25-85. No uptake was observed in low-grade gliomas. There were 4 lesions in 2 lymphoma cases included in this study. The TBR of [^68^Ga]Ga-FAPI was 67.5-78, which was better than other primary brain tumors on [^68^Ga]Ga-FAPI PET/CT. However, compared to [^18^F]F-FDG PET/CT, lymphomas showed a smaller and more heterogeneous uptake on [^68^Ga]Ga-FAPI PET/CT (**Figure 1**). The remaining 1 ependymoma, and 1 meningioma in this category also showed abnormal tracer concentration on [^68^Ga]Ga-FAPI PET/CT (SUVmax 1.6 VS. 2.5).

**Table 2:** [^68^Ga]Ga-FAPI and [^18^F]F-FDG uptake according to histological type.

Diagnosis	Number of lesions	[^68^Ga]Ga-FAPI		[^18^F]F-FDG	
		Median SUVmax (lesions)	Median TBR	Median SUVmax (lesions)	Median TBR
Primary brain tumors	18	1.8 (0.01-10.9)	45 (1-85.7)	11.4 (4.0-41.9)	1.9 (1-5)
Gliomas					
grade III-IV	9	1.5 (0.5-6)	40 (25-85.7)	11.1 (8.2-16.7)	1.8 (1.5-3.7)
grade I-II	3	0.04 (0.01-0.2)	1 (1-1)	5.6 (4.0-6.7)	1 (1-1)
Lymphomas	4	3.8 (2.6-10.9)	69.8 (54.5-78)	28.6 (28.5-41.9)	4.5 (4-5)
Ependymoma	1	1.6	53.3	6.3	1.5
Meningioma	1	2.5	6.3	12.1	1.9
Brain metastases	22	2.1 (0.01-17.0)	27.8 (1-230)	7.9 (2.0-17.9)	1.0 (1-2.1)
metastasis of lung cancer	18	2.3 (0.01-17.0)	27.8 (1-230)	8.0 (2.0-17.9)	1.1 (1-2.1)
metastasis of ovarian cancer	2	1.4 (0.07-2.8)	20.5 (1.4-40)	7.9 (7.4-8.3)	1.0 (1-1)
metastasis of thyroid cancer	1	0.9	45	6.8	1.0
metastasis of melanoma cancer	1	0.05	1	7.9	1.8

**Figure 2:**
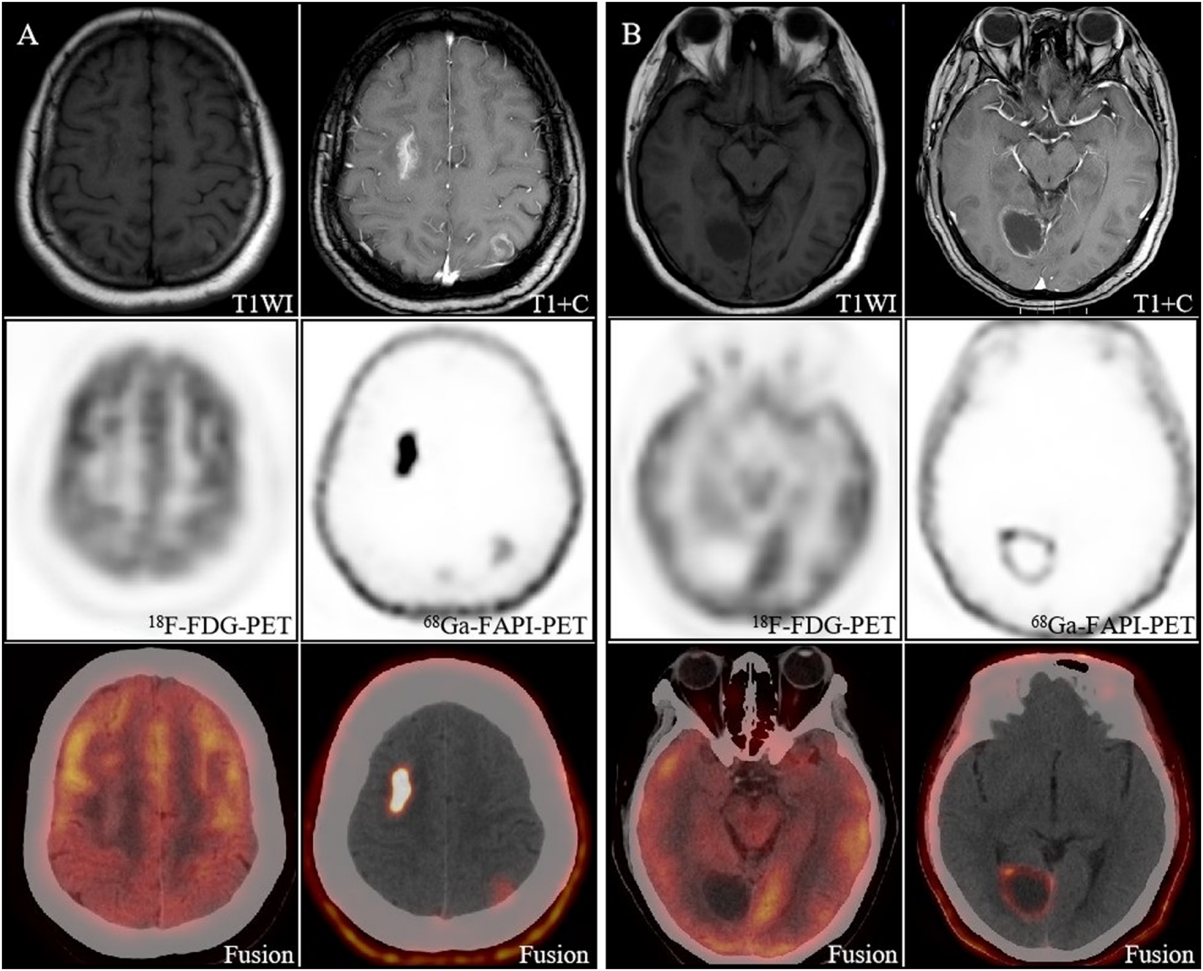
Image obtained in a 40-year-old woman with newly diagnosed lung cancer brain metastasis. **(A)** Brain metastasis in the right frontal lobe showed significant enhancement on MRI and showed intense [^68^Ga]FAPI activity (SUVmax 11.1). The lesion in the left parietal lobe showed ring-like mild enhancement on MRI and increased uptake on [^68^Ga]FAPI PET/CT (SUVmax 1.8). However, both lesions had no significant uptake on [^18^F]FDG PET/CT, and only the right frontal lobe lesion had morphological changes on CT. **(B)** The right occipital lobe lesion showed slight enhancement of the margins on MRI and increased uptake on [^68^Ga]FAPI PET/CT (SUVmax 4.8), which is still no uptake on [^18^F]FDG PET/CT.

In 22 brain metastases, 7 lesions showed no abnormal [^68^Ga]Ga-FAPI uptake. We observed that none of these 7 lesions had a maximum diameter of more than 1 cm, and even some of them had no morphological changes on CT and were confirmed as small metastases only on MRI. The SUVmax of [^68^Ga]Ga-FAPI was 0.01-11.1. On [^68^Ga]Ga-FAPI PET/CT, the lesion with the highest SUVmax was located in a patient with brain metastases from lung adenocarcinoma, and [^68^Ga]Ga-FAPI PET/CT revealed three lesions shown by MRI in this patient. Whereas only two of them were detected by [^18^F]F-FDG PET/CT, and none of them had [^18^F]F-FDG activity (**Figure 2**).

For [^68^Ga]Ga-FAPI PET/CT imaging, neither TBR nor SUVmax values differed significantly between primary brain tumors and brain metastases (P > 0.05). For [^18^F]F-FDG PET/CT imaging, TBR and SUVmax values were both higher in primary brain tumors than in brain metastases (P < 0.05).

### Patient-based analyses

The 13 primary brain tumors included 9 gliomas, 2 lymphomas, 1 ependymoma, and 1 meningioma. Of these, only 2 low-grade gliomas (grades I-II) were devoid of both FAPI and FDG activity, while the remaining 11 patients had increased uptake on both [^68^Ga]Ga-FAPI PET/CT and [^18^F]F-FDG PET/CT at sites where lesions were detected on MRI, and visual assessment showed good concordance between [^68^Ga]Ga-FAPI PET/CT and [^18^F]F-FDG PET/CT results.

**Figure 3:**
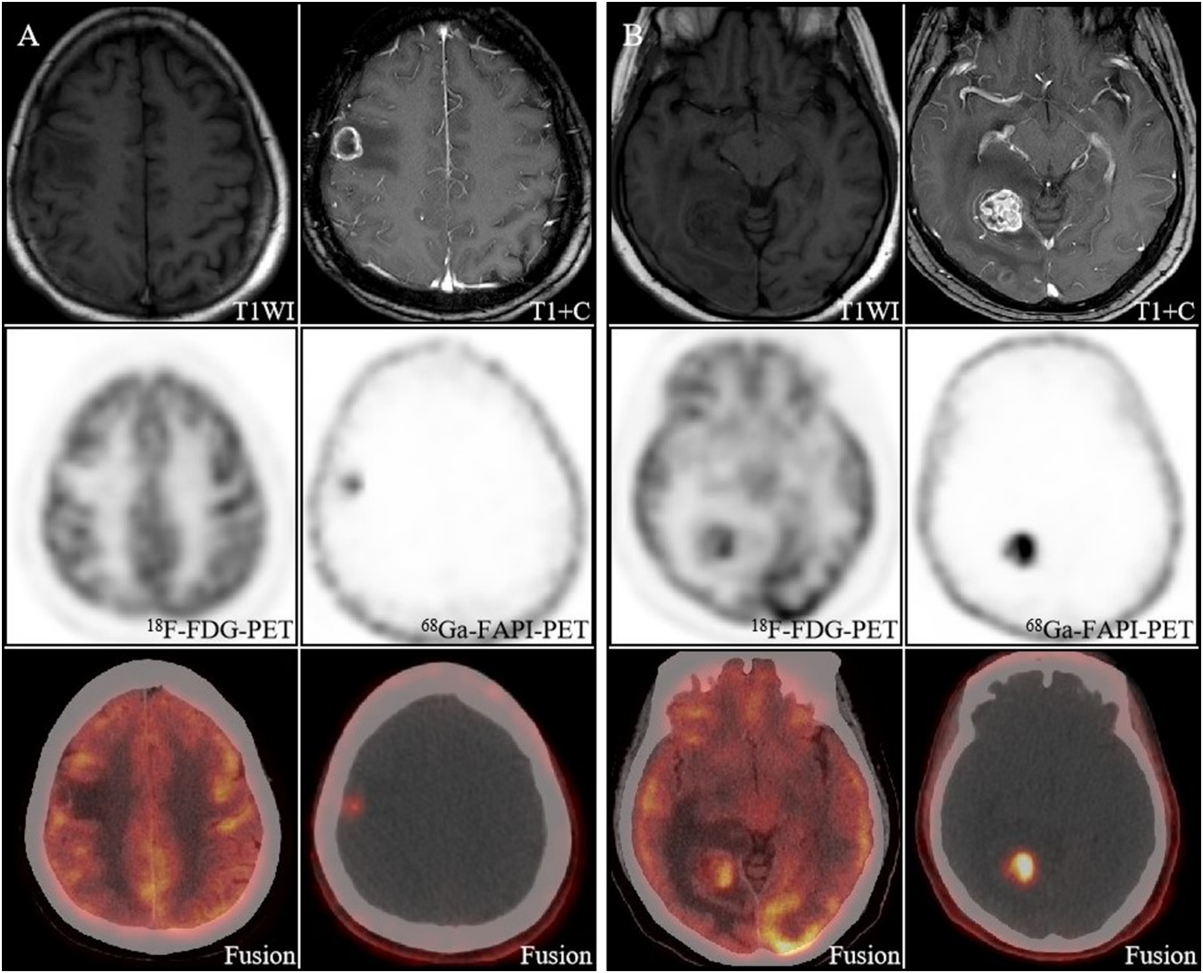
A 59-year-old woman with newly diagnosed brain metastases from lung cancer. Both the right parietal lobe lesion **(A)** and the right occipital lobe lesion **(B)** had morphological changes, with increased uptake of [^68^Ga]FAPI and a high TBR (38.3, 115), whereas on [^18^F]FDG PET/CT the lesions did not show as well as [^68^Ga]FAPI PET/CT.

Of the 12 brain metastases, 9 arose from lung cancer, 1 from ovarian cancer, 1 from thyroid cancer, and 1 from melanoma. 6 patients with brain metastases from lung cancer showed increased uptake of FAPI, whereas 7 showed abnormally increased FDG activity. However, [^68^Ga]Ga-FAPI PET/CT showed significantly better results than [^18^F]F-FDG PET/CT, even though the [^68^Ga]Ga-FAPI injection dose was lower. As shown in **Figure 3**, patients received [^18^F]F-FDG (203 Mbq) and [^68^Ga]Ga-FAPI (110 Mbq), but [^68^Ga]Ga-FAPI PET/CT significantly improved tumor visualization. In addition, in 1 ovarian cancer brain metastasis and 1 thyroid cancer brain metastasis, the lesion uptake of [^68^Ga]Ga-FAPI was increased but without [^18^F]F-FDG. In contrast, in patient with melanoma brain metastases, [^18^F]F-FDG was superior to [^68^Ga]Ga-FAPI.

### Relationship between lesion uptake of [^68^Ga]Ga-FAPI and various parameters of MRI with contrast

Of the 25 patients, 18 underwent MRI with contrast, with a total of 33 lesions (**Table 3**). Of these, 6 lesions exhibited no significant enhancement (fraction: 0). In addition, 13 lesions exhibited comparable or lower enhancement than the medulla oblongata (fraction: 1), and 14 exhibited significant enhancement (fraction: 2). All lesions had an edematous zone range of 0 - 4.7 cm and a maximum diameter range of 0.4 - 5.5 cm. Spearman’s correlation analyses indicated that SUVmax was only positively correlated with maximum diameter (P<0.001), whereas TBR was positively correlated with the degree of enhancement, edema zone range, and maximum diameter (P<0.05).

**Table 3:** Relationship between lesion uptake of [^68^Ga]Ga-FAPI and various parameters of MRI with contrast.

Lesion No.	Pathological type of primary tumor	[^68^Ga]Ga-FAPI		MRI		
		SUVmax	TBR	enhanced degree	edema zone (cm)	maximum diameter (cm)
1	Mucous ovarian adenocarcinoma	2.8	40	1	0.4	1.64
2	Mucous ovarian adenocarcinoma	0.07	1.4	0	0	0.53
3	Grade IV glioma	2.1	52.5	2	3	3.8
4	*Lung cancer	11.1	138.8	2	1	2.3
5	*Lung cancer	1.8	18	1	0	1.6
6	*Lung cancer	4.8	68.6	1	0	3.6
7	Grade IV glioma	1.2	30	2	1.6	5.5
8	Ependymoma	1.6	53.3	1	0	3.4
9	*Lung cancer	0.02	1	1	0	0.5
10	Small cell lung cancer	0.01	1	1	0	0.4
11	Small cell lung cancer	0.03	1	1	0	1.04
12	Small cell lung cancer	0.07	1	2	1.5	0.7
13	Grade IV glioma	1.5	50	2	2.8	3.5
14	*Lung cancer	3	37.5	2	4.7	3.0
15	Adenocarcinoma of lung	0.04	1	0	0	1.6
16	Grade IV glioma	0.7	35	2	1	2.24
17	Grade IV glioma	0.8	40	2	0.5	1.26
18	Grade IV glioma	0.5	25	2	0	1.36
19	*Lung cancer	2.3	38.8	2	1.9	1.6
20	*Lung cancer	4.6	115	2	2.6	2.2
21	Lymphoma	10.9	54.5	1	0	4.7
22	*Lung cancer	0.7	70	2	0	0.7
23	*Lung cancer	2.5	5	2	0	1.1
24	Adenocarcinoma of lung	4.3	61.4	1	3	2.8
25	Adenocarcinoma of lung	17	85	1	0	1.1
26	Adenocarcinoma of lung	3.7	7.4	0	0	1.0
27	Lymphoma	3.9	78	1	2	3.8
28	Lymphoma	2.7	67.5	1	0	2.2
29	Lymphoma	3.6	72	1	0	2.4
30	Grade II glioma	0.04	1	0	0	1.5
31	Grade II glioma	0.01	1	0	0	1
32	*Lung cancer	2.3	230	2	4.3	5.1
33	*Lung cancer	0.04	1	0	0	1.4

*Lung cancer means that there was no specific immunohistochemical classification.

Among the 33 lesions, 27 lesions had different degrees of enhancement, and 6 lesions had enhancement. The detection rate of [^68^Ga]Ga-FAPI PET for enhanced lesions was significantly higher than that for non-enhanced lesions (85.2% VS. 33.3%, P=0.007). The relationship between the detection of tumor by [^68^Ga]Ga-FAPI PET and the maximum diameter of tumor was further analyzed with the receiver operating characteristic (ROC). The optimal cut-off value was 1.62cm, the area under the curve (AUC) was 0.855 (P=0.003). That is, the maximum diameter of tumor ≥1.62cm was more likely to show increased uptake on [^68^Ga]Ga-FAPI PET.

## Discussion

Due to the unique intracranial blood-brain barrier (BBB) and tumor microenvironment, treatment outcomes for primary or metastatic brain tumors are poor compared to other solid or metastatic tumors. It is thus particularly important to find a suitable target for brain tumor imaging and therapeutic targeting. Brain tumor imaging techniques have evolved rapidly over the past few years, particularly with advances in MRI and PET.

MRI technology has continued to improve, and the use of diffusion-weighted imaging and wave analysis has grown more common, which allows for the clarification of the character of the lesion and its associated blood supply (49). These technologies have improved diagnostic efficacy for brain tumors and enabled more reliable grading of malignancy in addition to qualitative tumor diagnosis.

[^18^F]F-FDG and amino acid tracers are the predominant radiotracers used in brain tumor imaging (50). The physiological uptake of [^18^F]F-FDG by normal brain tissue and the effect of blood glucose result in a lower [^18^F]F-FDG PET/CT sensitivity for brain tumor detection. Amino acid PET/CT is the favored radiotracer for low-grade gliomas. It has also been shown that amino acid PET/CT exhibits accuracy superior to that of [^18^F]F-FDG PET/CT and MRI for outlining the extent of tumors (51). It is thus an essential tool for outlining the extent of tumors before surgery or radiotherapy for gliomas. However, due to the high cost of amino acid tracers and the increased uptake in the case of hematomas, it is rarely used clinically.

[^68^Ga]Ga-FAPI PET/CT has been studied widely in breast, lung, and pancreatic cancer imaging, and is particularly effective relative to [^18^F]F-FDG PET/CT for diagnosing gastrointestinal tumors and peritoneal diseases (52). FAP is also a target for brain tumor therapy because of its ability to promote tumor growth and invasion, inhibit tumor immune response, and induce temozolomide resistance (36). There are few imaging and therapeutic studies on this target in brain tumors.

In our cohort, the [^68^Ga]Ga-FAPI-derived TBR was particularly prominent, ranging from 1-230, with a median TBR of 39.9, significantly higher than the [^18^F]F-FDG-derived TBR of 1.5 (P < 0.001). Like many other intracranial tracers with no physiological uptake, such as [^18^F]F-FET and [^68^Ga]Ga-PSMA (53), the high TBR of [^68^Ga]Ga-FAPI PET/CT facilitates the detection of lesions and can accurately reveal even very small lesions. In addition, normal brain tissue exhibits minimal [^68^Ga]Ga-FAPI uptake, so FAP is very promising as a target for brain tumor therapy and may improve the survival rate of brain tumor patients. Study showed that [^177^Lu]Lu-FAP PTRT can be used more safely in the treatment of a wide range of cancers (54). However, a large number of future preclinical studies and human trials are needed to explore the value of FAP as a target in brain tumor therapy.

We evaluated 40 lesions in 25 patients and found that [^68^Ga]Ga-FAPI PET/CT outperformed [^18^F]F-FDG PET/CT in the detection of lesions (75% *vs*. 62.5%), particularly in the detection of metastatic brain tumors (63.6% *vs*. 45.5%). Detection of brain metastases is important for staging, treatment planning and prognosis. [^68^Ga]Ga-FAPI PET/CT will have an advantage over [^18^F]F-FDG PET/CT in evaluating systemic tumor brain metastases in patients who cannot receive MRI due to metal implants or claustrophobia. In addition, [^68^Ga]Ga-FAPI is cheaper than [^18^F]F-FDG (32).

While [^18^F]F-FDG PET/CT is not recommended for the detection of brain tumors, it is valuable for differentiating intracranial lymphomas from high-grade gliomas (55). It can also be used to assess the efficacy of treatment of intracranial lymphomas and differentiate between lymphoma recurrence and inflammatory lesions (56).

In this study, both TBR and SUVmax in primary brain tumors were elevated relative to those for brain metastases on [^18^F]F-FDG PET/CT, while this was not observed on [^68^Ga]Ga-FAPI PET/CT. This may be because this study included 2 diffuse large B lymphoma patients with a SUVmax range of 28.5-41.9 on [^18^F]F-FDG PET/CT, which is significantly higher than for other brain tumors. Previous studies have shown that a brain tumor with a TBR greater than 2.4 on [^18^F]F-FDG PETCT, allowing quantitative differentiation of gliomas and lymphoma (57). [^68^Ga]Ga-FAPI-derived TBR was higher than high-grade glioma in all lymphoma lesions. This indicates that [^68^Ga]Ga-FAPI PET/CT has the same potential as [^18^F]F-FDG PET/CT to differentiate the two. As glucose transporter proteins are overexpressed in both lymphoma cells and the tumor microenvironment, whereas FAP is expressed only in stromal cells, the uptake of FDG by lymphomas is greater than that of FAPI (58). Consistent with work by Xiao, the SUVmax range for lymphoma on [^68^Ga]Ga-FAPI PET/CT was 2.7-10.9 (59), which was lot lower than [^18^F]F-FDG PET/CT. It is worth noting that in this study, both the scope and degree of FAPI uptake within the lymphoma lesions are less than FDG, which may be related to less mesenchymal component and more lymphoma cells, which indicates a poor prognosis (60). Combination [^68^Ga]Ga-FAPI PET/CT and [^18^F]F-FDG PET/CT might be more helpful in prognostication of lymphoma patients.

Busek demonstrated that FAP was not expressed in normal brain tissue but was detectable in multiple components of the glioblastoma microenvironment and correlated with tumor grade (34). In our study, both grade III and IV gliomas took up [^68^Ga]Ga-FAPI and exhibited a good TBR, and there was no significant difference in their TBR, while both grade I and II tumors were negative for uptake. This is consistent with work by Rohrich et al. However, [^68^Ga]Ga-FAPI PET/CT is still unable to differentiate between grade III and IV gliomas. We believe that this may be because Grade III and Grade IV gliomas are both highly malignant tumors with no significant difference in damage to the blood-brain barrier, but the sample size is too small to further verify.

In this study, lesion size was strongly correlated with the degree of [^68^Ga]Ga-FAPI uptake (R = 0.61, P < 0.001). Our study showed that brain tumors with a maximum diameter > 1.62 cm were more likely to be detected by [^68^Ga]Ga-FAPI, while lesions < this value might be missed, which is the shortcoming of [^68^Ga]Ga-FAPI. This is likely because larger lesions are associated with a greater stromal volume. However, the uptake of [^68^Ga]Ga-FAPI by these lesions was not only dependent on the degree of FAP expression in the tumor stroma, but was also potentially limited by BBB permeability.

The BBB exists between systemic circulation and the brain. It blocks many molecules, including chemotherapy drugs, from entering the brain, contributing to poor outcomes for patients with intracranial tumors. On MRI with contrast, T1 tumor enhancement is a result of blood-brain barrier disruption and contrast leakage, while peritumoral edema of the tumor is also associated with BBB disruption. Therefore, the degree of T1 sequence enhancement and the extent of peritumoral edema can approximate the degree of BBB disruption (61).In our study, the degree of T1 tumor enhancement, the extent of the edema zone, and the uptake of [^68^Ga]Ga-FAPI by the target lesions were moderately positively correlated (0.40-0.44, P < 0.05). In addition, [^68^Ga]Ga-FAPI PET/CT had a significantly higher detection rate for enhanced lesions compared with non-enhanced lesions (85.2% VS. 33.3%, P=0.007). This confirms that the degree of BBB disruption is correlated with [^68^Ga]Ga-FAPI uptake. As such, [^68^Ga]Ga-FAPI PET/CT could be a potential imaging modality to respond to BBB permeability, and may not be suitable for evaluating lesions without enhancement on MRI.

There are limitations to these analyses. Firstly, the small sample size and the large variety of pathologies are not conducive to controlled analyses. In addition, IDH status is lacking for primary brain tumors and specific pathological types are lacking for brain metastases. Then, MRI with contrast can only approximate BBB permeability and cannot enable its quantitative assessment. Other MRI data such as DWI, DCE, Flair and so on, should be included in future studies and compared with [^68^Ga]Ga-FAPI uptake.

## Conclusion

[^68^Ga]Ga-FAPI PET/CT is a viable and potentially useful imaging tool for brain tumor assessment while also providing a basis for targeted therapy. Compared to [^18^F]F-FDG PET/CT, [^68^Ga]Ga-FAPI increases detection rate and offers a significantly improved TBR and can better visualize metabolically active lesions in the brain. It can be an advantageous supplement imaging for evaluation of lesions >1.5 cm and with BBB permeability.

## Data Availability

The original contributions presented in the study are included in the article/supplementary material. Further inquiries can be directed to the corresponding author.
